# Microbiota Profile and Impact of *Fusobacterium nucleatum* in Colorectal Cancer Patients of Barretos Cancer Hospital

**DOI:** 10.3389/fonc.2019.00813

**Published:** 2019-08-29

**Authors:** Ana Carolina de Carvalho, Leandro de Mattos Pereira, José Guilherme Datorre, Wellington dos Santos, Gustavo Noriz Berardinelli, Marcus de Medeiros Matsushita, Marco Antonio Oliveira, Ronilson Oliveira Durães, Denise Peixoto Guimarães, Rui Manuel Reis

**Affiliations:** ^1^Molecular Oncology Research Center, Barretos Cancer Hospital, Barretos, Brazil; ^2^Department of Pathology, Barretos Cancer Hospital, Barretos, Brazil; ^3^Epidemiology and Biostatistics Department, Barretos Cancer Hospital, Barretos, Brazil; ^4^Department of Medical Oncology, Barretos Cancer Hospital, Barretos, Brazil; ^5^Department of Prevention, Barretos Cancer Hospital, Barretos, Brazil; ^6^Life and Health Sciences Research Institute (ICVS), School of Medicine, University of Minho, Braga, Portugal; ^7^ICVS/3B's–PT Government Associate Laboratory, Braga, Portugal

**Keywords:** colorectal cancer, microbiota, *Fusobacterium nucleatum*, MSI, patient survival

## Abstract

Microbial diversity has been pointed out as a major factor in the development and progression of colorectal cancer (CRC). We sought to explore the richness and abundance of the microbial community of a series of colorectal tumor samples treated at Barretos Cancer Hospital, Brazil, through 16S rRNA sequencing. The presence and the impact of *Fusobacterium nucleatum* (*Fn*) DNA in CRC prognosis was further evaluated by qPCR in a series of 152 CRC cases. An enrichment for potentially oncogenic bacteria in CRC was observed, with *Fusobacterium* being the most abundant genus in the tumor tissue. In the validation dataset, *Fn* was detected in 35/152 (23.0%) of fresh-frozen tumor samples and in 6/57 (10.5%) of paired normal adjacent tissue, with higher levels in the tumor (*p* = 0.0033). *Fn* DNA in the tumor tissue was significantly associated with proximal tumors (*p* = 0.001), higher depth of invasion (*p* = 0.014), higher clinical stages (*p* = 0.033), poor differentiation (*p* = 0.011), MSI-positive status (*p* < 0.0001), BRAF mutated tumors (*p* < 0.0001), and the loss of expression of mismatch-repair proteins MLH1 (*p* < 0.0001), MSH2 (*p* = 0.003), and PMS2 (*p* < 0.0001). Moreover, the presence of *Fn* DNA in CRC tissue was also associated with a worse patient cancer-specific survival (69.9 vs. 82.2% in 5 years; *p* = 0.028) and overall survival (63.5 vs. 76.5%; *p* = 0.037). Here we report, for the first time, the association of *F. nucleatum* presence with important clinical and molecular features in a Brazilian cohort of CRC patients. Tumor detection and classification based on the gut microbiome might provide a promising approach to improve the prediction of patient outcome.

## Introduction

Colorectal cancers (CRCs) are the third most incident tumors worldwide and the second in mortality rates, with almost 2 million new cases and over 881,000 deaths estimated to occur in 2018 (1). The increased incidence of CRC in the last decades can be explained by population aging, poor dietary habits, and lifestyle factors such as smoking, low physical activity, and obesity (2, 3). Around 55% of the cases occur in more-developed regions of the world, with more than half of the deaths occurring in less-developed regions, reflecting a worse prognosis in these areas (4). The improvements in survival in developed countries have been achieved through the adoption of best practices in cancer treatment and management (3).

In Brazil, CRC is the second most frequent type of cancer in women and the third most frequent in men (5). A recent study observed an increase in rate of mortality from CRC in Brazil, for both men and women when comparing data from 1996 to 2012 (6). These tumors accounted for 5.1% of all death by cancer in men in 1996 and for 6.9% in 2012; for women, the rates increased from 6.9% in 1996 to 8.2% in 2012 (6). Therefore, although the incidence of these tumors in Brazil remains lower than that of developed, high-income countries, mortality rates are similar, reflecting the disparity in the mortality–incidence ratio occurring in Brazil (7, 8).

Diagnosis, prognosis, and therapy choice depend on tumor classification and staging, which are based on morphology and histology (9, 10) However, despite an increasing understanding of the pathophysiology of CRC in the past decades, the introduction of additional methods that could be accurately used for screening, risk prediction, prognosis, choice of treatment, and monitoring in a clinical setting has been challenging.

The majority of CRC cases have a sporadic nature (11). Genetic, epigenetic, and environmental factors play an important role in the etiology of CRC through oncogene activation and tumor suppressor genes silencing, therefore contributing to the acquisition of hallmark cancer traits in colon epithelial cells (12). Several studies have been exploring the influence of microbial diversity in the initiation and progression of these tumors (13, 14). Several findings point to changes in the composition and activity of the gut microbiome, creating a microenvironment that promotes inflammation, proliferation, and neoplastic progression as a result of an interplay between these events, host genetics, and other environmental factors (15, 16). Some bacteria have been reported to be enriched or diminished in CRC (17). *Fusobacterium nucleatum* (*Fn*) strains are the most consistently reported to promote carcinogenesis when successful in invading host cells (18–20). However, the extent of CRC cases that can be attributed to these agents, how their abundance can affect the gut microbiome, and how they can be used in a clinical setting in cancer screening, treatment, or management remains unclear.

Tumor detection and classification based on the gut microbiome might provide a promising approach to improve early diagnosis and the prediction of patient outcome. Therefore, this study unraveled the richness and abundance of the microbial community of colorectal tumor samples in comparison to healthy mucosa through 16S rRNA gene sequencing in a small cohort of patients treated at Barretos Cancer Hospital, and correlated different microbial phylotypes with clinicopathologic features and molecular characteristics (such as tumor location, *BRAF* mutation, and MSI status). Furthermore, following the observed enrichment of members of the *Fusobacterium* genus in CRC cases, we sought to evaluate the presence of *Fn* in tumor samples and adjacent normal mucosa. Besides confirming higher rates of this bacteria in tumor samples, we also observed significant associations with important patient clinical features, suggesting the oncogenic role of this bacteria in CRC carcinogenesis.

## Materials and Methods

### Patient Population and Tissue Samples

This study analyzed samples from 152 patients with CRC treated surgically at the Department of Colorectal Surgery of Barretos Cancer Hospital, Barretos, Brazil between 2008 and 2015. Patients were followed for a median of 59.68 months (ranging from 2.37 to 104.97 months). All samples used were collected from the surgical resection material. Clinical and pathological characteristics of patients, such as age, gender, location of primary tumor, staging, and histological grade, as well as molecular data on the expression of mismatch repair proteins (MLH1, MSH6, MSH2, and PMS2), status of molecular microsatellite instability (MSI) (21), and *BRAF* mutation (unpublished data) were available from previous studies from our group.

Fresh-frozen tumor tissue was available for 152 cases and paired formalin-fixed paraffin-embedded (FFPE) for 139, while normal adjacent fresh-frozen tissue was available for a subset of 57 cases. Tumor tissue and normal adjacent mucosa were snap-frozen immediately following excision of the specimen at surgery and stored at −80°C at the Barretos Cancer Hospital Biobank until processing. Slides from all tissue specimens were carefully micro-dissected and subjected to histological examination to confirm the diagnostic as normal or cancerous tissue. Only tumor samples with the presence of at least 60% of tumor cells were included. DNA from fresh-frozen tissue samples was extracted using the DNA Mini Qiasymphony kit (Qiagen, Valencia, CA), while DNA from FFPE samples was extracted using the QIAamp DNA Micro Kit (Qiagen, Valencia, CA) as previously reported (22).

This study was approved by the Institutional Review Board at Barretos Cancer Hospital (Project number 1402/2017).

### 16S rRNA Gene Sequencing

Fifty nanograms of genomic DNA from 15 paired fresh-frozen tumor and normal adjacent samples were used to generate libraries of the V4 region of the 16S rRNA (515F 5′-GTGCCAGCMGCCGCGGTAA-3′, 806R 5′-GGACTACHVGGGTWTCTAAT-3′) (23) using the Fusion PCR primer technology for templated bead preparation. Genomic DNA targets amplification was carried out according to the manufacturer's instructions using Platinum PCR SuperMix High Fidelity polymerase (Invitrogen, Carlsbad, California) and bar-coded primers (Invitrogen, Carlsbad, California) at an annealing temperature of 56°C for 40 cycles. PCR products were cleaned up with Agencourt AMPure XP Kit Purification system (Beckman Coulter, Brea, USA), quantified by Qubit, and multiplexed at equimolar concentrations of 40 pM. Template preparation and loading into Ion 314 v2 Chips (Life Technologies, Carlsbad, California) was conducted using the Ion Chef System (Life Technologies, Carlsbad, California) workflow and sequencing was performed in an Ion Personal Genome Machine (PGM) System using the Ion PGM Hi-Q View Chef Kit (Life Technologies, Carlsbad, California). Bioinformatics analysis of 16S sequencing is described in Supplementary Methods. The data that support the findings of this study are openly available in BioProject under sequence accession number PRJNA543496.

### Quantitative PCR (qPCR) for *Fn*

Genomic DNA obtained from 152 tumor and 57 normal adjacent fresh-frozen tissue samples and from 139 FFPE tumor samples were subjected to quantitative PCR using TaqMan primer-probe sets (Applied Biosystems) for the 16S ribosomal RNA gene DNA sequence of *Fn* (*nusG*), and for the reference gene, *SLCO2A1* as previously described (24).

The primer and probe sequences used were as follows: *nusG* forward primer, 5′-CAACCATTACTTTAACTCTACCATGTTCA-3′; *nusG* reverse primer, 5′-GTTGACTTTACAGAAGGAGATTATGTAAAAATC-3′; *nusG* probe, 6FAM-GTTGACTTTACAGAAGGAGATTA-MGBNFQ; *SLCO2A1* forward primer, 5′-ATCCCCAAAGCACCTGGTTT-3′; *SLCO2A1* reverse primer, 5′-AGAGGCCAAGATAGTCCTGGTAA-3′; *SLCO2A1* probe, 6FAM-CCATCCATGTCCTCATCTC-MGBNFQ.

Each reaction contained 100 ng of genomic DNA run in duplicate in 20-μL reactions containing 1 × final concentration TaqMan Environmental Master Mix 2.0 (Applied Biosystems), 900 nM primers and 500 nM probes for each target gene. Amplification and detection were performed with the QuantStudio 6 Flex Real-Time PCR System (Thermo Fisher Scientific) using the following reaction conditions: 10 min at 95°C and 45 cycles of 15 s at 95°C and 1 min at 60°C. DNA from *F. nucleatum* subsp. *nucleatum Knorr* (ATCC 2558) was kindly provided by Prof. Mario Julio Avila Campos (USP, SP, Brazil) and used as positive control for all *nusG* runs.

In colorectal carcinoma cases with detectable *Fn*, the cycle threshold (Ct) values in the quantitative PCR for *nusG* were normalized by *SLCO2A1* and used to calculate 2^−Δ*Ct*^ values that were used to quantify the amount of *Fn* DNA in each sample as a relative unitless value (where ΔCt = the average Ct value of *nusG*—the average Ct value of *SLCO2A1*) as previously described (25). These samples were classified according to the amount of bacteria found as low or high (*Fn-low, Fn-high)*, on the basis of the median cut point amount of *Fn* DNA in all samples with positive results (tumor or normal adjacent), according to sample storage method (fresh-frozen median = 4.5 × 10^−6^, FFPE median = 6.0 × 10^−6^), as previously described (26).

### Statistical Analysis

Statistical analysis was performed using the software IBM SPSS Statistics 21 for Windows. Categorical variables were compared using Fisher's exact test. For all analysis, we considered statistical significance when *p* ≤ 0.05 (two-sided).

We conducted univariate logistic regression analyses to assess associations of the amount of tissue *Fn* DNA as a three-category variable (*Fn-negative, Fn-low, Fn-high*) with the following variables, using the negative status as a reference: tumor location, the extent of the tumor into the wall of the colon or rectum (T), tumor differentiation, MSI status, and *BRAF* mutations. The multivariable logistic regression model initially included age (continuous), sex, tumor location (proximal colon vs. distal or rectum), MSI (positive vs. negative), *BRAF* (mutant vs. wild type), and MLH1, MSH2, MSH6, and PMS2 protein expression (negative vs. positive). A backward stepwise elimination with a threshold of *p* = 0.05 was used to select variables in the final models. To assess independent associations of MSI and tumor location (predictor variables) with the amount of tissue *Fn* DNA (an ordinal outcome variable; negative vs. low vs. high), we performed univariate ordinal logistic regression analysis.

To test for associations between the presence of *Fn* DNA with overall mortality and cancer-specific mortality, we classified it into two categories: cases with detectable *Fn* DNA (*Fn* positive) and cases without detectable *Fn* DNA (*Fn* negative). Kaplan–Meier curves were constructed and the log-rank test was used to assess differences in mortality between the two categories. The Cox proportional hazards regression model was used to control for confounders. Multivariable models included age (continuous), sex, tumor location (proximal colon vs. distal or rectum), MSI (positive vs. negative), *BRAF* (mutant vs. wild type), and MLH1, MSH2, MSH6, and PMS2 protein expression (negative vs. positive). A backward stepwise elimination with a threshold of *p* = 0.05 was used to select variables in the final models.

## Results

### Microbial Diversity in Tumor and Normal Tissues

The microbiota profile of 15 paired tumor and normal adjacent tissues from patients with colorectal carcinoma was evaluated by next-generation sequencing of V4 variable region of 16S rRNA. A total of 2,198,654 good quality reads with a mean length of 269 base pairs were generated. A total of 41,844 OTUS comprising all 30 samples (based on ≥99% of identity) were generated with the UPARSE algorithm. The community structure represented by diversity or richness was evaluated (from 0 to 30,000 reads) using classical ecological indexes of alpha-diversity (Observed OTUS, Chao1, PD_whole_tree, and Shannon Index) and by constructing a rarefaction curve until both curves tended to reach a plateau. Only nine CRC and normal tissue pairs reached the plateau at 30,000 reads (Supplementary Figures 1A,B) with an average of 1,406 OTUS in the tumor and 1,412 OTUS in the normal tissue. The bacterial community of nine paired tumor and normal adjacent tissues from this cohort was characterized. The clinical and molecular characteristics of this subset of CRC patients is presented in Supplementary Table 1. These samples were used for downstream analyses of the taxonomic profile and differential abundance. The results of alpha diversity metrics (Observed OTUs, Chao1, and Shannon) after rarefaction with 30,000 reads/samples of depth are also shown in boxplots of Supplementary Figure 1C. No significant difference in alpha diversity between the groups tested at the highest rarefaction depths (30,000 reads/samples) was observed (ANOVA and *t*-test; *p* > 0.05), suggesting that tumor and normal tissue collected from the same patients have similar diversity of species and richness.

The Unifrac unweighted beta diversity analysis also showed that the overall bacterial community structure and phylogenetic diversity in tumor and normal samples tested were similar, as distinct clusters could not be observed for the two sample types. The same analysis showed that the most abundant genus (Enterotype) involved in clustering this samples in similar groups is represented by *Fusobacterium, Bacteroides*, and *Escherichia* (Supplementary Figure 2).

### Taxonomic Profiling of Microbiota in Tumor and Normal Tissues

**Figure 1:**
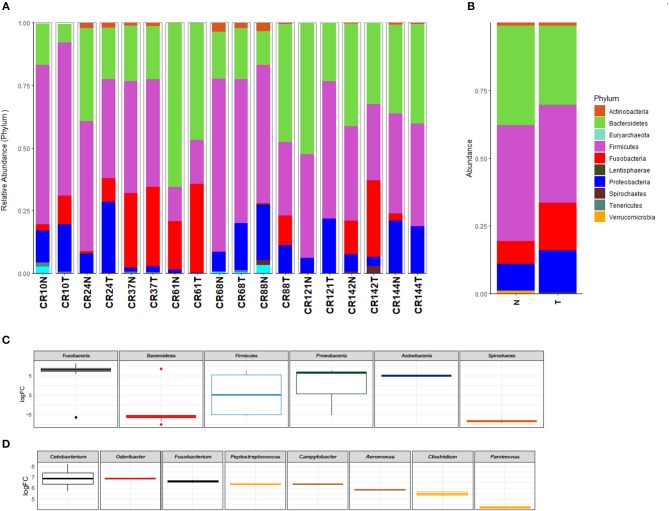
Taxonomic profile of the microbiota at phylum (>0.1%) level identified in colorectal carcinoma and normal adjacent mucosa pairs of samples **(A)** and grouped by sample type (tumor vs. normal) **(B)**. Differential abundance between tumor and normal tissue in log fold change (LogFC), showing significantly increased phylum **(C)** and genus **(D)** in colorectal carcinoma in comparison to normal tissue (*p* < 0.05). T, colorectal carcinoma; N, normal adjacent mucosa.

The analysis of the taxonomic profile at the phylum level in tumor (T) and normal adjacent (N) tissue identified Actinobacteria, Bacteroidetes, Euryarchaeota, Firmicutes, Fusobacteria, Lentisphaerae, Proteobacteria, Spirochaetes, Tenericutes, and Verrucomicrobia as the most frequent (Figures 1A,B). The evaluation on paired tumor and normal tissue from the same patient revealed that 6/9 sample (CR10N: 2.4% vs. CRC10T: 11.2%, CR24N: 0.7% vs. CR24T: 9.4%, CR61N: 19.3% vs. CR61T: 35.5%, CR88T: 0.2% vs. CR88N: 11.5%, CR142N:13.3% vs. CR142T: 30.7%) were enriched in Fusobacteria in the CRC tissue and 5/9 sample (CR10N: 12.7% vs. CRC10T: 19%, CR24N: 7.8% vs. CR24T: 28.6%, CR68N: 7.8% vs. CR68T: 18.7%, CR121N: 6.4% vs. CR121T: 27.4%) had an increase of Proteobacteria (Figure 1A). We also analyzed the taxonomic profile of all samples grouped by sample type (tumor vs. normal) (Figure 1B).

At the family level, 30 abundant members were identified in tumor and normal samples (Supplementary Figure 3). When results from the samples evaluated were grouped into tumor and normal adjacent, Fusobacteriaceae (N: 7.5% vs. T: 15.8%), Enterobacteriaceae (N: 6.7% vs. T: 8.8%), and Streptococcaceae (N: 1.8% vs. T: 4.0%), were more enriched in the tumor tissue. Finally, at the genus level, the most abundant genera identified in the tumor tissue are described in Supplementary Figure 4. *Fusobacterium* (N: 2.9% vs. T: 7.9%), *Streptococcus* (N: 1.8% vs. T: 4.0%), *Parvimonas* (N: 1.0% vs. T: 1.8%), *Aeromonas* (N: 0.0% vs. T: 1.0%), *Campylobacter* (N: 0.0% vs. T: 0.8%), *Cetobacterium* (N: 0.0% vs. T: 0.7%), and *Clostridium* (N: 0.0% vs. T: 0.5%) were most overrepresented.

The fluctuation and density of population frequencies of *Fusobacterium* in CRC tumor (and normal adjacent tissue) were evaluated using the two-dimensional kernel density tool (2D-kde). This evaluation was able to reveal a gradual increase of abundance (log10 transformed relative abundance) of the *Fusobacterium* population from normal to tumor tissue, with a higher density peak of the *Fusobacterium* population in tumor (Supplementary Figure 5).

### Phylum and Genera Abundance in Tumor and Normal Tissues

The analysis of the log2 fold-change values of the differential abundance between tumor and normal tissue showed that the most significantly enriched phylum in CRC samples evaluated were Fusobacteria, Proteobacteria, and Actinobacteria (Figure 1C). The log2 fold-change values distribution of differential abundance between tumor and normal tissue showed that the most enriched genera in CRC is constituted by *Cetobacterium, Odoribacter, Fusobacterium, Peptostreptococcus, Campylobacter, Aeromonas, Clostridium*, and *Parvimonas* (Figure 1D); all these genera were identified with a *p*-value below 0.05, while the genera *Pseudomonas, Ruminococcus, Prevotella, Paraprevotella, Leptotrichia, Bacteroides*, and *Treponema* were found depleted in CRC (Supplementary Figure 6).

### *Fn* in Tumor and Normal Tissues

Since *Fusobacterium* genus was overexpressed in tumor tissue, we further evaluated the presence of a fragment of the 16S ribosomal RNA gene from *Fn* in tumor and normal adjacent samples.

**Figure 2:**
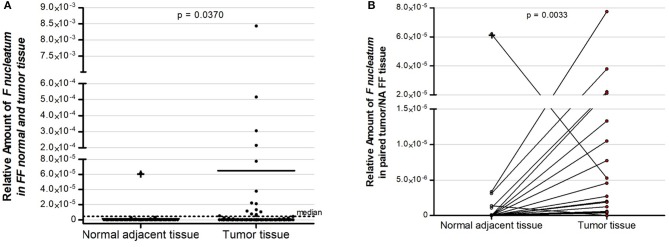
Relative amount of *F. nucleatum* in colorectal cancer patients: **(A)** Fifty seven normal adjacent fresh-frozen tissue samples and 152 colorectal fresh-frozen carcinoma tissue samples. Dot plots represent samples and the dotted line represents the median cut point amount (median = 4.5 × 10^−6^) around which samples were classified as having high (above median) or low (below median) amount of *F. nucleatum*. **(B)** Overrepresentation of *F. nucleatum* in colorectal carcinoma tissue samples in comparison to normal adjacent tissue in 57 paired cases. Statistical analysis was performed using independent Mann–Whitney test **(A)** and paired Wilcoxon signed rank test **(B)**. FF, fresh–frozen; NA, normal adjacent.

Firstly, we evaluated tumor tissue of 152 fresh-frozen CRC cases, and in a subset of 57 cases, we also assessed normal adjacent tissue. *Fn* DNA was detected in 35 (23.0%) of the 152 colorectal carcinoma samples and in 6 (10.5%) of the 57 normal adjacent tissue samples evaluated (Figure 2A; independent two-tailed Mann–Whitney test, *p* = 0.0370). In the 57 pairs of colorectal carcinoma and normal adjacent tissues, the amount of *Fn* DNA was significantly higher in colorectal carcinoma tissue than in the normal adjacent tissue, except for one patient with proximal colon, MSI-positive and *BRAF* mutated tumor, in which the level of *Fn* DNA was 11.88-fold higher in the normal-adjacent tissue (Figure 2B, paired two-tailed Wilcoxon signed rank test, *p* = 0.0038).

In the same cases, we further evaluated *Fn* in FFPE tumor tissue. In the 139 colorectal carcinoma samples available to be tested, *Fn* DNA was detected in only 8 (5.8%) of cases. There was an absence of correlation between *Fn* detection between results obtained from fresh-frozen tissue and FFPE samples (Cohen's Kappa test = 0.167; Supplementary Table 2).

The 35 fresh-frozen and eight FFPE CRC cases with detectable *Fn* DNA were classified using the median cut point amount (fresh-frozen median = 4.5 × 10^−6^, FFPE median = 6.0 × 10^−6^) into two groups according to the level of *Fn* DNA: high (above median) or low (below median) (Supplementary Tables 3, 4).

### Association of *Fn* With Clinical–Pathological and Molecular Features and Patient Survival

Clinical, demographic, and molecular data of the 152 CRC patients evaluated are presented in Table 1. The mean age at diagnostics was 60.63 (±13.7) years and 53.3% of the patients profiled in this cohort were male. Regarding the primary tumor site, most of the tumors were in the distal colon (75.0%), were classified according to tumor stage as T3/T4 (65.1%) and with EII/EIII clinical stage (28.9% of E0/1; 67.1% of EII/EIII; and 3.9% EIV), and were well to moderately differentiated histologically (92.7%) (Table 1). Results of MSI status (21), *BRAF* mutation (unpublished data), and expression of mismatch-repair proteins MLH1, MSH2, MSH6, and PMS2 (21) were obtained from other studies from our group and are also described in Table 1. Most of the patients were MSI-negative (92.7%), with MLH1, MSH2, MSH6, and PMS2 expression (87.3, 97.8, 99.3, and 88.8%, respectively).

**Table 1:** Clinical, pathological, and molecular features according to the amount of *Fusobacterium nucleatum* (Fn) in fresh-frozen colorectal carcinoma tissue.

**Variables**	**All cases, *n* (%)**	**Amount of** ***Fn*** **in tumor tissue (*****n*** **=** **152)**			
		***Fn-neg*, *n* (%), *n* = 117**	***Fn-low*, *n* (%), *n* = 17**	***Fn-high*, *n* (%), *n* = 18**	***p*-value^†^**
Mean age (years) ± SD	60.63 ± 13.7				
**Gender**					
Female	71 (46.7)	53 (45.3)	7 (41.2)	11 (61.1)	0.406
Male	81 (53.3)	64 (54.7)	10 (58.8)	7 (38.9)	
**Tumor location**					
Proximal colon	38 (25.0)	21 (17.9)	7 (41.2)	10 (55.6)	0.001
Distal colon or rectum	114 (75.0)	96 (82.1)	10 (58.8)	8 (44.4)	
**Tumor (T)**					
Tis/T1/T2	53 (34.9)	47 (40.2)	2 (11.8)	4 (22.2)	0.035
T3/T4 (a b)	99 (65.1)	70 (59.8)	15 (88.2)	14 (77.8)	
**Clinical stage**					
E0/I	44 (28.9)	39 (33.3)	2 (11.8)	2 (16.7)	0.089
EII/III	102 (67.1)	74 (63.2)	13 (76.5)	15 (83.3)	
EIV	6 (3.9)	4 (3.4)	2 (11.8)	0 (0.0)	
**Tumor differentiation**					
Well to moderate	139 (92.7)	110 (94.0)	17 (100.0)	12 (75.0)	0.011
Poor	11 (7.3)	7 (6.0)	0 (0.0)	4 (25.0)	
**MSI status^#^**					
MSI-negative	131 (86.2)	110 (94.0)	11 (64.7)	10 (55.6)	<0.0001
MSI-positive	21 (13.8)	7 (6.0)	6 (35.3)	8 (44.4)	
***BRAF*** **mutation**^**$**^					
Mutant	11 (7.3)	3 (2.6)	3 (17.6)	5 (27.8)	<0.0001
Wild type	140 (92.7)	113 (97.4)	13 (76.5)	14 (77.8)	
**MLH1 protein expression^#^**					
Positive	117 (87.3)	101 (95.3)	8 (61.5)	8 (53.3)	<0.0001
Negative	17 (12.7)	5 (4.7)	5 (38.5)	7 (46.7)	
**MSH2 protein expression^#^**					
Positive	131 (97.8)	105 (99.1)	11 (84.6)	11 (84.6)	0.003
Negative	3 (2.2)	1 (0.9)	2 (15.4)	2 (15.4)	
**MSH6 protein expression^#^**					
Positive	133 (99.3)	105 (99.1)	13 (100.0)	15 (100.0)	0.875
Negative	1 (0.7)	1 (0.9)	0 (0.0)	0 (0.0)	
**PMS2 protein expression^#^**					
Positive	119 (88.8)	102 (96.2)	9 (69.2)	8 (53.3)	<0.0001
Negative	15 (11.2)	4 (3.8)	4 (30.8)	7 (46.7)	

*Percentage indicates the proportion of cases with a specific clinical, pathological, or molecular variable according to the amount of F. nucleatum DNA in colorectal cancer tissue*.

† *To assess associations between the ordinal categories (negative, low, and high) of the amount of F. nucleatum DNA in colorectal cancer tissue and categorical variables, Fisher's exact test was performed*.

# Previously reported by Berardinelli et al. (21);

$ *Unpublished data; MSI, microsatellite instability*.

The association between clinical, pathological, and molecular characteristics and the levels of *Fn* DNA detected (high, low, or negative) in fresh-frozen tissue are summarized in Table 1. For FFPE, results are summarized in Supplementary Table 4.

The amount of *Fn* DNA in fresh-frozen CRC tissue was associated with proximal tumor location (*p* = 0.001), higher depth of invasion (*p* = 0.035), poorly differentiated tumors (*p* = 0.011), MSI-positive (*p* < 0.0001), *BRAF* mutated tumors (*p* < 0.0001), and with the loss of expression of mismatch-repair proteins MLH1 (*p* < 0.0001), MSH2 (*p* = 0.003), and PMS2 (*p* < 0.0001).

Either low, high, or both categories of *Fn* DNA positivity showed a significant impact on all clinical, pathological, and molecular data tested, as measured by univariate odds ratio (OR) models adjusted by logistic regression (Table 2). In multivariable logistic regression analyses, the amount of tissue *Fn* DNA was statistically associated with MSI status: Fn-low had an OR of 6.812 (95% CI, 1.544 to 30.051; *p* = 0.011) for MSI-positive, while *Fn-high* had an OR of 7.206 (95% CI, 1.769–29.357; *p* = 0.006). Proximal tumors were also significantly associated with MSI-positive status, with an OR of 16.685 (95% CI, 4.779–58.250; *p* < 0.0001). For the other variables tested in the univariate analysis, no statistically significant association was observed with tissue *Fn* DNA in the multivariable analyses.

**Table 2:** Univariate odds ratio (OR) models adjusted by logistic regression of the impact of the amount of *Fusobacterium nucleatum* (*Fn*) in colorectal cancer tissue and clinical, pathological, and molecular data.

**Variables**	**Univariable OR (95% CI)**	***p*-value**
**Proximal vs. distal colon**		
*Fn-negative*	1 (reference)	
*Fn-low*	3.20 (1.27–9.38)	0.034
*Fn-high*	5.71 (2.01–16.2)	0.001
**T3/T4 vs. Tis/T1/T2**		
*Fn-negative*	1 (reference)	
*Fn-low*	5.04 (1.10–23.05)	0.037
*Fn-high*	2.35 (0.73–7.58)	0.153
**Poor vs. well to moderate**		
*Fn-negative*	1 (reference)	
*Fn-low*	Not evaluated	Not evaluated
*Fn-high*	5.24 (1.34–20.5)	0.017
**MSI-positive vs. MSI-negative^#^**		
*Fn-negative*	1 (reference)	
*Fn-low*	8.57 (2.45–30.05)	0.001
*Fn-high*	12.57 (3.77–41.88)	<0.0001
**Mutated vs. wild type** ***BRAF***^$^		
*Fn-negative*	1 (reference)	
*Fn-low*	8.07 (1.48–43.92)	0.016
*Fn-high*	14.49 (3.10–67.72)	0.0001
**Negative vs. positive MLH1^#^**		
*Fn-negative*	1 (reference)	
*Fn-low*	12.63 (3.01–52.94)	0.001
*Fn-high*	17.68 (4.56–68.50)	<0.0001
**Negative vs. positive MSH2^#^**		
*Fn-negative*	1 (reference)	
*Fn-low*	19.09 (1.60–227.86)	0.02
*Fn-high*	Not evaluated	Not evaluated
**Negative vs. positive PMS2^#^**		
*Fn-negative*	1 (reference)	
*Fn-low*	11.33 (2.41–53.10)	0.002
*Fn-high*	22.31 (5.37–92.65)	<0.0001

*MSI, microsatellite instability; CI, confidence interval; OR, odds ratio*.

# Previously reported by Berardinelli et al. (21);

$ *unpublished data*.

We also performed an OR model adjusted by ordinal logistic regression analysis to assess the impact of MSI status, tumor extension through the wall (T), tumor location, differentiation, status of *BRAF* mutation, and MLH1, MSH2, and PMS2 protein expression in the amount of tissue *Fn* DNA. Only T3/T4 tumor stage (OR, 2.81; 95% CI, 1.04–7.55; *p* for trend 0.028) and MSI-positive (OR, 8.64; 95% CI, 3.33–22.39; *p* < 0.0001) were associated with the amount of tissue *Fn* DNA (Table 3).

**Table 3:** Ordinal logistic regression analysis to assess associations of tumor stage and MSI status with the amount of *Fusobacterium nucleatum* DNA in colorectal cancer tissue.

**Variables**	**Odds ratio (95% CI)**	***p*-value**
***Fn-negative*** **vs**. ***Fn-low*** **vs**. ***Fn-high***		
Tumor stage (T3/T4 vs. Tis/T1/T2)	2.81 (1.04–7.55)	0.028
MSI (MSI-positive vs. MSI-negative)^#^	8.64 (3.33–22.39)	<0.0001

*MSI, microsatellite instability; CI, confidence interval*.

# *Previously reported by Berardinelli et al. (21)*.

**Figure 3:**
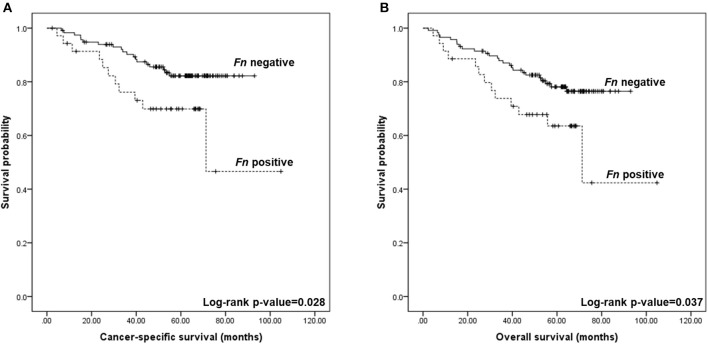
Kaplan–Meier curves for colorectal cancer-specific and overall survival according to the detection of *Fusobacterium nucleatum* (*Fn*) DNA in colorectal tissue. **(A)** Five-year cancer-specific survival was of 69.9% for *Fn* positive and 82.2% for *Fn* negative (log-rank *p* = 0.028). **(B)** Five-year overall survival was of 63.5% for *Fn* positive and 76.5% for *Fn* negative (log-rank *p* = 0.037).

Regarding patient outcome, the presence of *Fn* DNA in CRC fresh-frozen tissue was associated with shorter cancer-specific survival (69.9 vs. 82.2% at 5 years; log-rank *p* = 0.028; Figure 3A) and overall survival (63.5% vs. 76.5% at 5 years; log-rank *p* = 0.037; Figure 3B). Multivariable hazard ratio for cancer-specific mortality in *Fn* positive cases was 2.255 (95% CI, 1.071–4.747; *p* = 0.032), and that for overall survival was 2.011 (95% CI, 1.028–3.937; *p* = 0.041).

In FFPE CRC tissue, the amount of *Fn* DNA was associated with proximal tumor location (*p* = 0.009), MSI-positive (*p* < 0.0001), *BRAF* mutated tumors (*p* = 0.002), and the loss of expression of mismatch-repair proteins MLH1 (*p* < 0.0001) and PMS2 (*p* < 0.0001). No further analyses were performed for these cases, as the number of positive cases was very low.

## Discussion

The human microbiome represents a complex ecosystem consisting of a large number of microorganisms that interact with the environment and host (27–30) with accumulating evidence showing their potential role in the pathogenesis of various neoplasms including CRC (14, 31–34).

This study firstly unveiled the richness and abundance of the microbial community of colorectal tumor samples in comparison to adjacent normal mucosa through 16S rRNA gene sequencing in a small cohort of Brazilian patients. Beta diversity analyses showed a similar structure of phylogenetic diversity of the bacterial community in CRC when compared to normal tissue from the same patient, and similar results have already been described in other studies (35, 36). However, it also indicated a fine-tuning transition of abundance of specific genera between the healthy microbiome present in normal tissue to potential oncogenic associated bacteria in CRC. Our study identified an increase of Fusobacteria and Proteobacteria phyla in CRC samples, which has been previously associated with dysbiosis, inflammation, and CRC (35). At the family level, the Enterobacteriaceae, Fusobacteriaceae, and Streptococcaceae families were distinctly enriched in CRC. Furthermore, we identified a core of microbiome enriched in CRC constituted by genus *Cetobacterium, Odoribacter, Fusobacterium, Peptostreptococcus, Campylobacter, Aeromonas, Clostridium*, and *Parvimonas*. Consistent with our findings, several others studies have reported *Fusobacterium* enrichment in human CRC tissue in comparison with adjacent normal tissue (37, 38). The combined population frequencies of *Fusobacterium* coupled to Kernel density analysis revealed a fluctuating variation in abundance of this genus from normal to CRC represented by a rise and peaks of abundance in CRC.

Therefore, despite the complex profile of changes in the balance of the intestinal microbiota, different metagenomic studies have shown an enrichment of the genus *Fusobacterium*, and most frequently the species *Fn* in adenomas and colorectal carcinomas in comparison with normal adjacent mucosa samples (18, 24, 37). The potential use of these microbes as non-invasive biomarkers for the detection of CRC has been explored (39, 40). Recently, Dai et al. conducted a comprehensive meta-analysis of shotgun metagenomics on CRC (41). The authors explored changes in gut microbiome that were universal across populations, by combining metagenomic data from 526 samples from Chinese, Austrian, American, German, and French cohorts, and found seven bacteria, including *Fn*, that were enriched in CRC across these populations. These bacteria were able to accurately classify cases (AUC = 0.80) across the different populations, demonstrating a potential of bacterial markers as robust diagnostic markers across populations (41).

The role of *Fn* in colonic carcinogenesis has been frequently implicated with progression of advanced colorectal carcinoma, and for this reason, this species has been mostly investigated as a prognostic factor. Previous studies have suggested that this species promotes colon carcinogenesis through the inhibition of proliferation and induction of apoptosis in T cells (18). Other studies revealed that elevated levels of *Fn* in the colon tissue are inversely correlated with the density of T CD3+ cells and strongly associated with MSI and CPG methylator phenotype (42, 43). Given these complex interactions between these microorganisms, immunity, genetic profile, and CRC, this study evaluated the abundance of *Fn* in DNA samples from CRC tissue through quantitative PCR, and the association and impact of this species in patient clinical and molecular features.

A recent study conducted a systematic review of all original scientific articles published between 2000 and 2017 investigating *Fusobacterium* and its relationship with CRC. After reviewing the 90 articles retrieved, the prevalence of *Fn* DNA in CRC tissue varied between 8.6 and 87.1%. The authors suggest that the wide variability observed could be explained by heterogeneity in study design, sampling, analysis methodology, population, geographic location, or diet (44). A recent study using samples from Brazilian CRC patients detected *Fn* DNA in 33/43 (76.7%) of CRC samples and also a very high rate of detection of *Fn* DNA in their paired normal adjacent mucosa (31/43; 72.1%) (45). However, the association between the presence of this bacteria and clinical information was not evaluated in the Brazilian population.

Our study found a prevalence of 23% of *Fn* DNA in (35/152) fresh-frozen CRC samples and of 10.5% (6/57) in the normal adjacent tissue samples. The presence of *Fn* in fresh-frozen tumor was associated with key clinical and molecular features of CRC: proximal tumor location, higher depth of invasion, higher clinical stages, poorly differentiated tumors, MSI-positive status, *BRAF* mutated tumors, and the loss of MMR proteins, here represented by the lack of IHC expression. Besides, either low, high, or both categories of *Fn* DNA positivity showed a significant impact on all clinical, pathological, and molecular data tested, as measured by univariate odds ratio (OR) models adjusted by logistic regression. In the multivariable model, together with proximal tumors, *Fn* low or high was associated with MSI-positive status. The presence of *Fn* was also associated with a higher cancer-specific mortality and lower overall survival. These results agree with findings from previous studies conducted in other populations, suggesting a role of *Fn* with a subtype of more aggressive CRC and a worse prognosis (42, 43, 46–49).

A much lower rate of positivity for *Fn* DNA was observed in FFPE samples (5.8%), with a low concordance with fresh-frozen results. Mima et al. previously acknowledged the limitations of using FFPE tissue to detect microorganisms, since routine histopathology procedures such as tissue fixation, paraffin embedding, and storage may influence qPCR assay results (43). Adding to this, it is known that in formalin-fixed tissue, cross-linking of histone-like proteins to DNA or fragmentation/degradation of genomic DNA occurs over time, further decreasing the sensitivity of identifying organisms (50); however, FFPE samples may be the only type of sample available for testing. For this reason, we tested for associations of the presence of *Fn* DNA in FFPE CRC and clinical and molecular data and found similar results as already reported in previous studies using both fresh-frozen and FFPE (42, 43, 46–49).

Along with the literature, our data reinforce specific organisms as components of a microbiome core present in CRC tissues. We observed that *Fn* is also found enriched in cohorts of patients from Brazil and that the presence of this bacteria is very likely to contribute to tumor aggressiveness and to a poor prognosis. A more in-depth study of the association between the metabolism of *Fusobacterium* and CRC can reveal the biological role and involved factors in tumor progression and may lead to personalized treatments.

In conclusion, we believe that tumor classification based on the gut microbiome might provide a promising approach to improve prediction of patient outcome. Despite the average sample size of this study, we were able to validate features of the association between the detection of *Fn* DNA and CRC carcinogenesis that are already consolidated in other populations. The findings presented here need to be validated in larger cohorts of Brazilian patients to assess if this species can be used as a marker in the Brazilian population, allowing a better management of patient prognostication.

## Data Availability

The datasets generated for this study can be found in the BioProject–/PRJNA543496.

## Author Contributions

AdC participated in study design, processed samples, carried out the microbiome and *Fn* detection experiments, analyzed the data, and prepared the manuscript. LdM participated in study design, carried out the bioinformatics analysis, and prepared the manuscript. JD processed samples and carried out *Fn* detection experiments. WdS processed samples, collected clinical data, and carried out the molecular characterization of samples. GB processed samples, collected clinical data, and carried out the molecular characterization of samples. MM performed the histopathological evaluation of all samples. MO performed the statistical analysis. RD collected clinical data. DG participated in study design and coordination. RR participated in study design and coordination and prepared the manuscript. All authors read and approved the final manuscript.

### Conflict of Interest Statement

The authors declare that the research was conducted in the absence of any commercial or financial relationships that could be construed as a potential conflict of interest.
