# Supplementary material for: Microbiota Profile and Impact of Fusobacterium nucleatum in Colorectal Cancer Patients of Barretos Cancer Hospital
Source: Front Oncol. 2019 Aug 29;9:813. doi: 10.3389/fonc.2019.00813 (PMC6727361; doi:10.3389/fonc.2019.00813)
Supplement: Supplementary file 1 [file Data_Sheet_1.docx]

**Supplementary Figures**


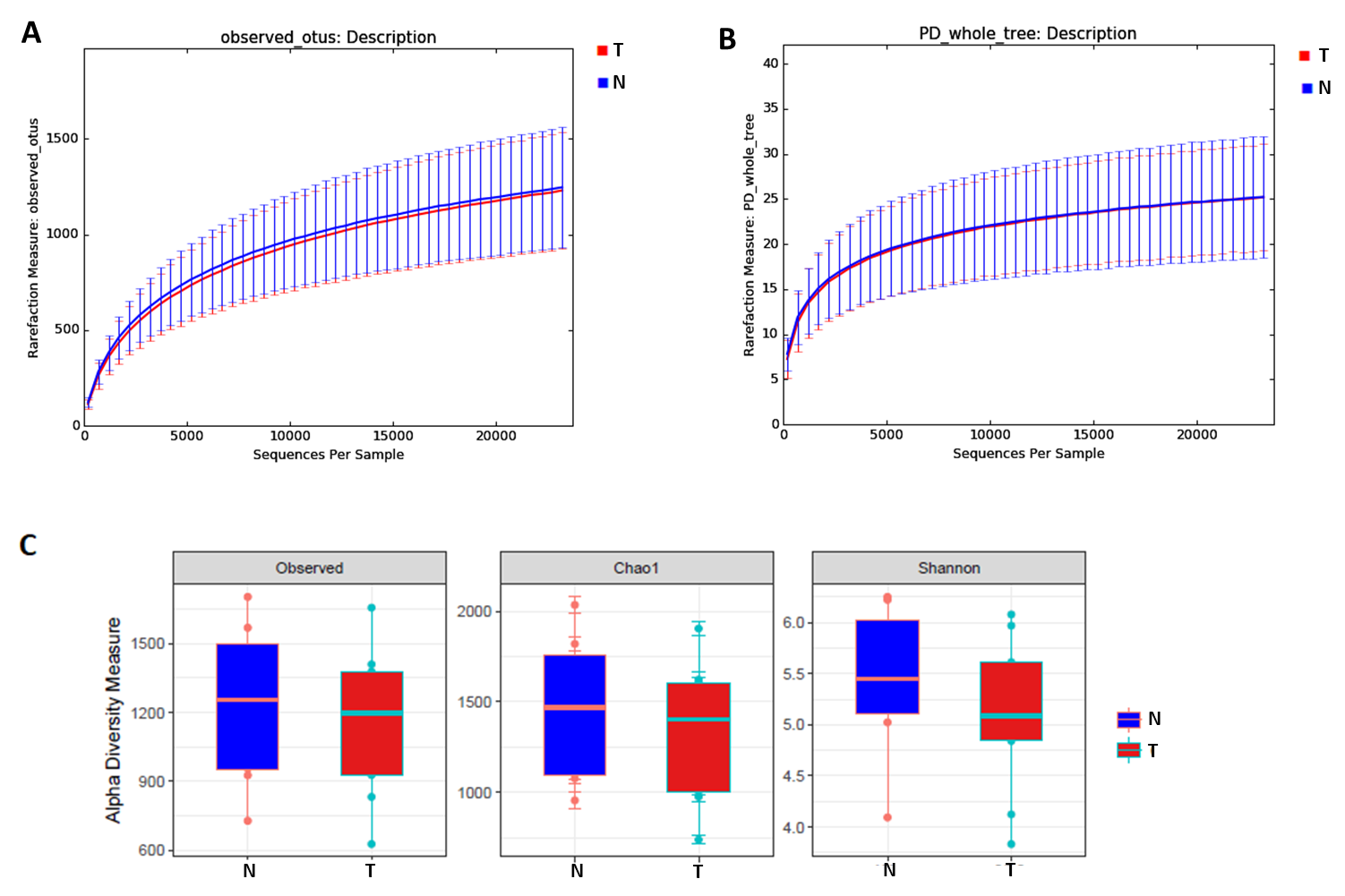


**Supplementary Figure 1 - Community structure evaluated in 9 paired CRC and normal tissue samples.** **A.** Rarefaction curve showing the average number of observed OTUS for both groups. **B.** Rarefaction curve showing the average number of phylogenetic diversities by metric Faith's phylogenetic diversity whole tree (PD_whole_tree) for both groups. **C.** Boxplots showing alpha-diversity using different metrics (Observed OTUs, Chao1 and Shannon index). Error bars represent standard error of the mean. T, colorectal tumor tissue; N, normal tissue.


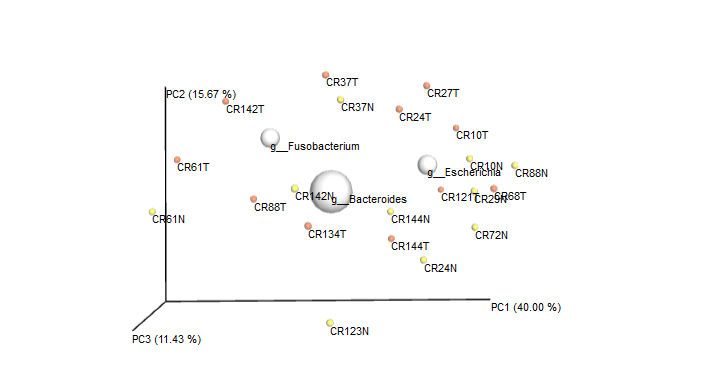


**Supplementary Figure 2 -** Clustering of paired tumor (T) and normal adjacent (N) samples from CRC patients based on Principal Coordinate Analysis (PCoA) using Unweighted UniFrac. White balls represent the centering of the most frequent genera plotted using Biplots on the basis of summarized taxa table generated on QIIME v1.8.0. Red dots represent CRC samples; Yellow dots represent NA samples. PCoA 3D plots were visualized using EMPeror.


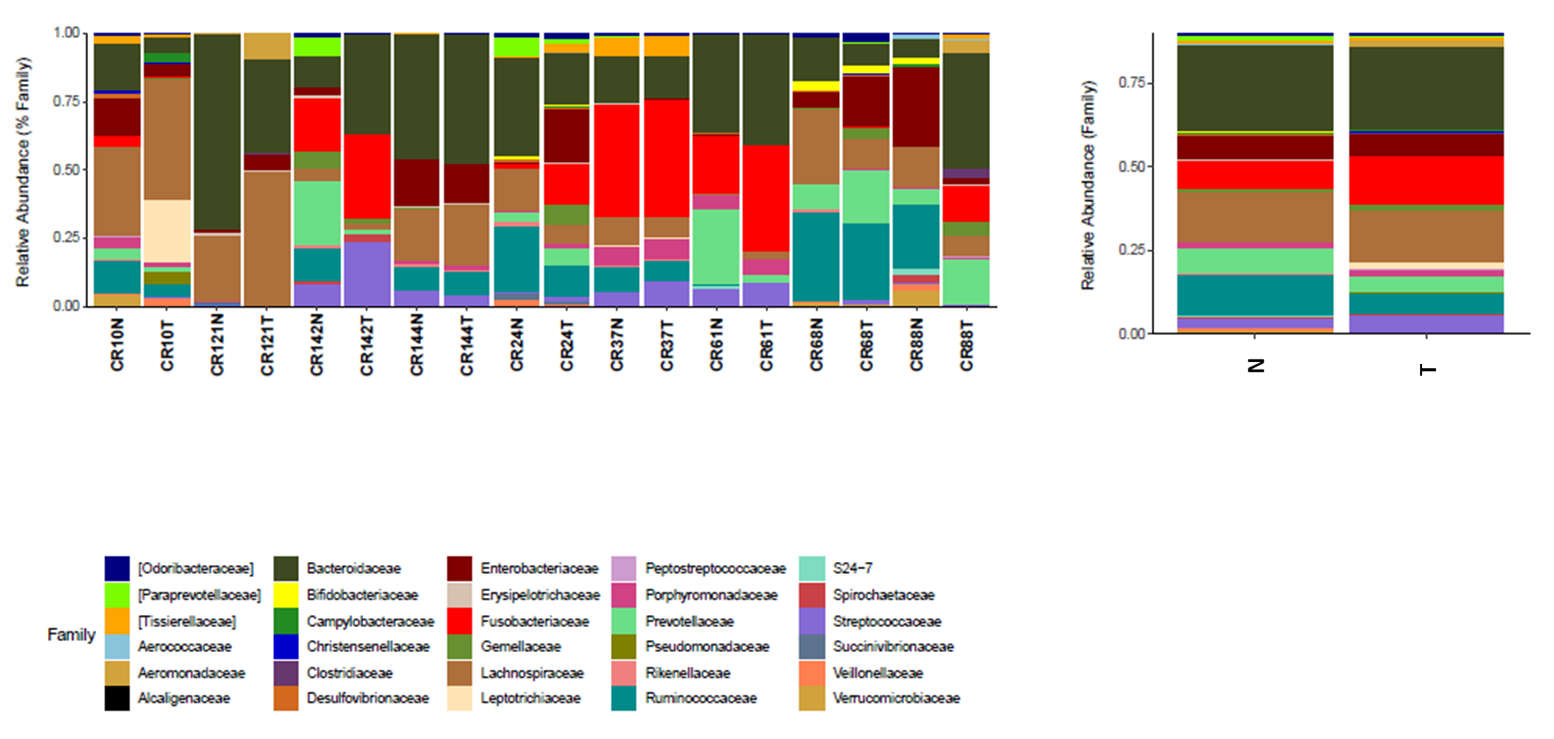


**Supplementary Figure 3 -** Taxonomic Profile of the Microbiota at Family level (>0.1%) identified in colorectal carcinoma (T) and adjacent normal tissue (N).


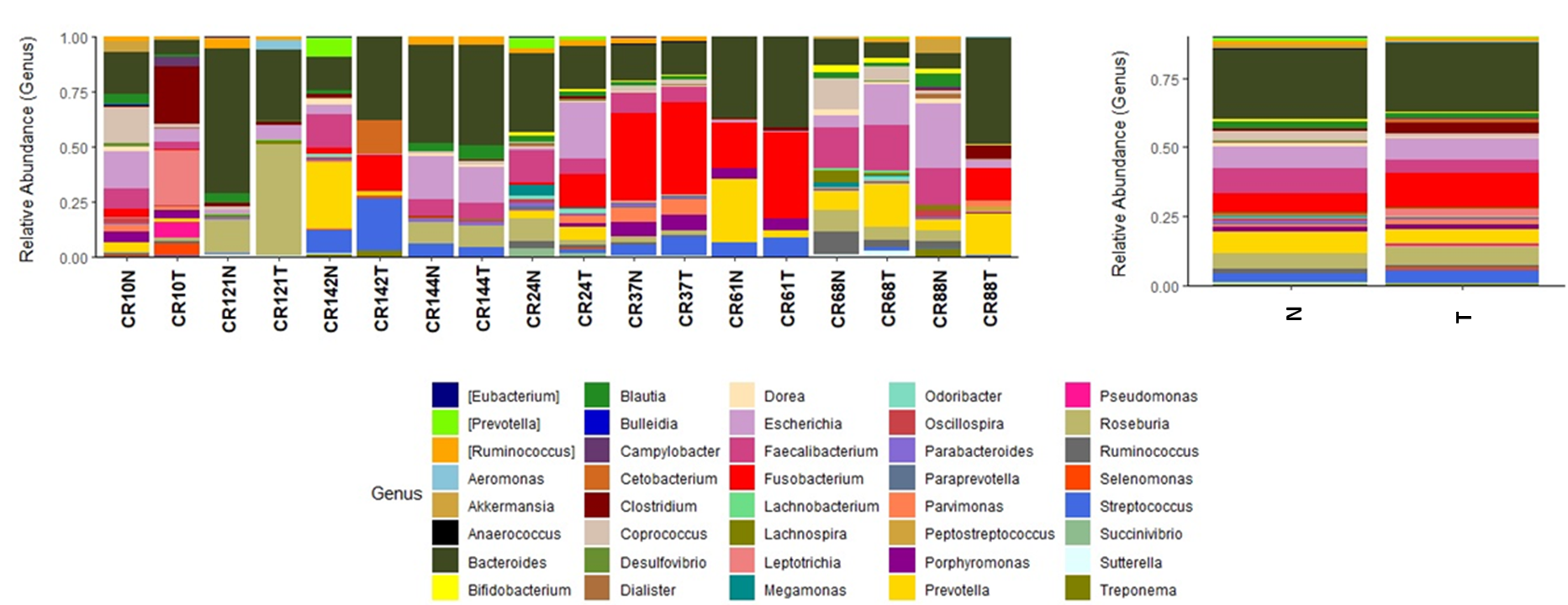


**Supplementary Figure 4 -** Taxonomic Profile of the Microbiota at Genus level identified in colorectal carcinoma (T) and adjacent normal tissue (N).


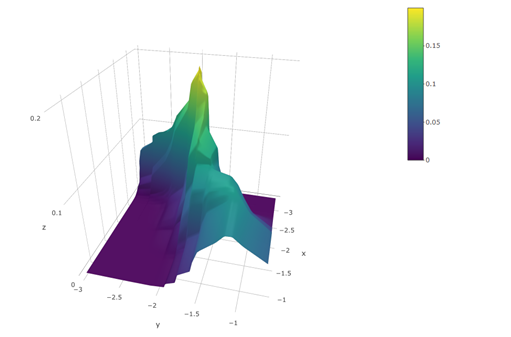


**Supplementary Figure 5 -** Microbiome Landscape of *Fusobacterium*. Two-dimensional kernel density (2D-kde) estimates showing the abundance of *Fusobacterium* (log10 transformed relative abundance data) in CRC and NA tissue. The z-axis represents the kernel density estimate in a 3D perspective plot showing the population frequencies of *Fusobacterium* in CRC (y-axis) and normal (x-axis) tissue. A gradual increase in abundance of this population from normal to CRC samples is observed.

**
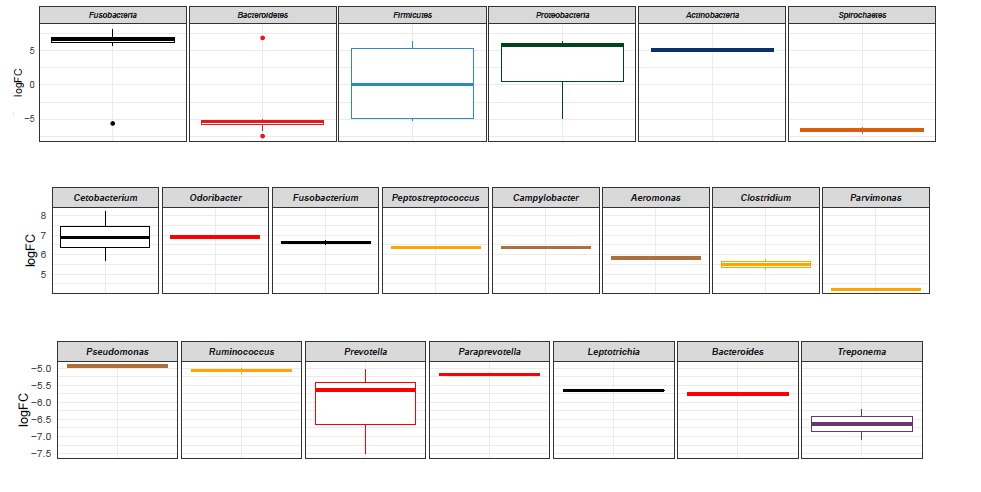
**

**Supplementary Figure 6** - Differential abundance between colorectal carcinoma (T) and adjacent normal tissue (N) in log fold-change (LogFC). Genus significantly decreased (p<0.05) in CRC.
