# Supplementary material for: Microbiota Profile and Impact of Fusobacterium nucleatum in Colorectal Cancer Patients of Barretos Cancer Hospital
Source: Front Oncol. 2019 Aug 29;9:813. doi: 10.3389/fonc.2019.00813 (PMC6727361; doi:10.3389/fonc.2019.00813)
Supplement: Supplementary file 2 [file Data_Sheet_2.docx]

**Supplementary Method - Bioinformatics analysis of 16S Metabarcoding**

After sequencing, the raw sequencing reads of 16S variables regions were submitted to quality control checking that remove sequences with lengths less than 100 base pairs and with less than 30 of Phred scale score using PRINSEQ (PReprocessing and INformation of SEQuences) ([Schmieder and Edwards, 2011](#_ENREF_9)). The remaining sequences were de-replicates, sorted in descending order of abundance of reads and clustered in operational taxonomic units (OTUS) using a 99% identity value through the V-SEARCH ([Rognes et al., 2016](#_ENREF_8)), according to the algorithm UPARSE ([Edgar, 2013](#_ENREF_3)). The chimeras were removed using *RDP gold* database (<https://drive5.com/uchime/gold.fa>). The taxonomic assignment was obtained using QIIME v1.8 ([Caporaso et al., 2010](#_ENREF_1)) through the RDP Naive Bayesian Classifier algorithm ([Wang et al., 2007](#_ENREF_12)) with 0.8 of score confidence using the GreenGenes 13.8 database ([DeSantis et al., 2014](#_ENREF_2)). The rarefaction curve and the alpha and beta diversity analyses were performed using QIIME (software v1.8) and R packages phyloseq ([McMurdie and Holmes, 2013](#_ENREF_6)) and the results were plotted with ggplot2 ([Wickham, 2009](#_ENREF_13)). For the alpha diversity analysis, the ecological index Observed OTUS, Chao1 and PD Whole tree were applied and were performed with QIIME v1.8 ([Caporaso et al., 2010](#_ENREF_1)). The number of OTUs was calculated with biom summarize-table of the Biom Project ([McDonald et al., 2012](#_ENREF_5)). The normalization and differential abundance was assessed for tumor and normal samples using the DESeq2 method incorporated through of phyloseq-to-deseq tool within the Phyloseq package ([McMurdie and Holmes, 2013](#_ENREF_6)). In brief, after TMM normalization ([Pereira et al., 2018](#_ENREF_7)) a negative binomial model was used to identified the differential abundance of genus in the samples that reached the plateau (30.000 sequences/samples) of the rarefaction curve. For the analysis of the beta diversity, which makes the comparison among the samples, we used the UNIFRAC unweighted measure accommodating the multivariate statistical technique PCoA Jackknifing ([Lozupone et al., 2011](#_ENREF_4)). The alpha-diversity comparison between treatments was performed through T-test and Anova. The analysis of landscaping of *Fusobacterium* in CRC and NA samples was performed with densities analysis using Kernel density estimation with the kde2d function implemented in MASS ([Venables et al., 2002](#_ENREF_11)) R package. For this analysis the relative frequency of OTUS in *Fusobacterium* in the CRC and NA samples were calculated and transformed into log10 (abundance) and used for density estimation using kde2d function. The 3D figure was generated with R package Plotly ([https://CRAN.R-project.org/package=plotly](https://cran.r-project.org/package=plotly)). This analysis was based on a previous study which analyzed the landscaping of microbiomes in Western adult population ([Shetty et al., 2017](#_ENREF_10)). The data that support the findings of this study are openly available in BioProject under sequence accession number PRJNA543496.

**References**

Caporaso, J.G., Kuczynski, J., Stombaugh, J., Bittinger, K., Bushman, F.D., Costello, E.K., et al. (2010). QIIME allows analysis of high-throughput community sequencing data. *Nat Methods* 7(5)**,** 335-336. doi: 10.1038/nmeth.f.303.

DeSantis, C.E., Lin, C.C., Mariotto, A.B., Siegel, R.L., Stein, K.D., Kramer, J.L., et al. (2014). Cancer treatment and survivorship statistics, 2014. *CA Cancer J Clin* 64(4)**,** 252-271. doi: 10.3322/caac.21235.

Edgar, R.C. (2013). UPARSE: highly accurate OTU sequences from microbial amplicon reads. *Nat Methods* 10(10)**,** 996-998. doi: 10.1038/nmeth.2604.

Lozupone, C., Lladser, M.E., Knights, D., Stombaugh, J., and Knight, R. (2011). UniFrac: an effective distance metric for microbial community comparison. *ISME J* 5(2)**,** 169-172. doi: 10.1038/ismej.2010.133.

McDonald, D., Clemente, J.C., Kuczynski, J., Rideout, J.R., Stombaugh, J., Wendel, D., et al. (2012). The Biological Observation Matrix (BIOM) format or: how I learned to stop worrying and love the ome-ome. *Gigascience* 1(1)**,** 7. doi: 10.1186/2047-217X-1-7.

McMurdie, P.J., and Holmes, S. (2013). phyloseq: an R package for reproducible interactive analysis and graphics of microbiome census data. *PloS one* 8(4)**,** e61217. doi: 10.1371/journal.pone.0061217.

Pereira, M.B., Wallroth, M., Jonsson, V., and Kristiansson, E. (2018). Comparison of normalization methods for the analysis of metagenomic gene abundance data. *BMC Genomics* 19(1)**,** 274. doi: 10.1186/s12864-018-4637-6.

Rognes, T., Flouri, T., Nichols, B., Quince, C., and Mahe, F. (2016). VSEARCH: a versatile open source tool for metagenomics. *PeerJ* 4**,** e2584. doi: 10.7717/peerj.2584.

Schmieder, R., and Edwards, R. (2011). Quality control and preprocessing of metagenomic datasets. *Bioinformatics* 27(6)**,** 863-864. doi: 10.1093/bioinformatics/btr026.

Shetty, S.A., Hugenholtz, F., Lahti, L., Smidt, H., and de Vos, W.M. (2017). Intestinal microbiome landscaping: insight in community assemblage and implications for microbial modulation strategies. *FEMS Microbiol Rev* 41(2)**,** 182-199. doi: 10.1093/femsre/fuw045.

Venables, W.N., Ripley, B.D., and Venables, W.N.M.a.s.w.S.P. (2002). *Modern applied statistics with S.* New York: Springer.

Wang, Q., Garrity, G.M., Tiedje, J.M., and Cole, J.R. (2007). Naive Bayesian classifier for rapid assignment of rRNA sequences into the new bacterial taxonomy. *Appl Environ Microbiol* 73(16)**,** 5261-5267. doi: 10.1128/AEM.00062-07.

Wickham, H. (2009). *ggplot2 : elegant graphics for data analysis.* New York ; London: Springer.
