# Supplementary material for: Microbiota Profile and Impact of Fusobacterium nucleatum in Colorectal Cancer Patients of Barretos Cancer Hospital
Source: Front Oncol. 2019 Aug 29;9:813. doi: 10.3389/fonc.2019.00813 (PMC6727361; doi:10.3389/fonc.2019.00813)
Supplement: Supplementary file 3 [file Data_Sheet_3.docx]

**Supplementary Tables**

**Supplementary Table 1.** Clinical and molecular data of CRC patients evaluated in the sequencing analysis.

| Sample ID | Gender | Age (years) | Tumor side | Clinical stage | MSI status**^#^** | *BRAF* mutation**^$^** |
| --- | --- | --- | --- | --- | --- | --- |
| CR_010 | Male | 58 | Distal colon | IIC | negative | No |
| CR_024 | Female | 67 | Distal colon | IIC | negative | No |
| CR_037 | Female | 81 | Distal colon | IIA | negative | No |
| CR_061 | Female | 42 | Distal colon | IIA | negative | No |
| CR_068 | Female | 39 | Distal colon | 0 | negative | No |
| CR_088 | Male | 72 | Proximal colon | I | positive | No |
| CR_121 | Female | 86 | Proximal colon | I | positive | No |
| CR_142 | Male | 75 | Proximal colon | IIA | positive | No |
| CR_144 | Female | 72 | Proximal colon | IIIB | negative | No |

^MSI=Microsatellite instability; # previously reported Berardinelli et al. 2019; $ unpublished data^

**Supplementary Table 2.** Concordance of *Fusobacterium nucleatum* (*Fn*) detection and level in FFPE and fresh-frozen samples from CCR cases.

|  |  |  | **FFPE n (%)** | | |
| --- | --- | --- | --- | --- | --- |
|  |  | ***Fn-negative*** | ***Fn-low*** | ***Fn-high*** | **Cohen´s Kappa** |
| **Fresh-frozen n (%)** | ***Fn-negative*** | 104 (99.0) | 0 (0.0) | 1 (1.0) | 0.167 |
|  | ***Fn-low*** | 15 (88.2) | 1 (5.9) | 1 (5.9) |  |
|  | ***Fn-high*** | 13 (76.5) | 2 (11.8) | 2 (11.8) |  |

FFPE=formalin-fixed paraffin embedded

**Supplementary Table 3.** Clinical, pathological and molecular characteristics and association with the amount of *Fusobacterium nucleatum (Fn)* in FFPE Colorectal Carcinoma Tissue.

|  |  | **Amount of Fn in FFPE tumor tissue (n=139)** | | | |
| --- | --- | --- | --- | --- | --- |
| **Variables** | **All cases n (%)** | **Fn-neg n (%) n=132** | **Fn-low n (%) n=3** | **Fn-high n (%) n=4** | **p-value†** |
| **Mean age (years) ±SD** | 60.63 ± 13.7 |  |  |  |  |
|  |  |  |  |  |  |
| **Gender** |  |  |  |  |  |
| **Female** | 71 (46.7) | 63 (47.7) | 2 (66.7) | 0 (0.0) | 0.133 |
| **Male** | 81 (53.3) | 69 (52.3) | 1 (33.3) | 4 (100.0) |  |
|  |  |  |  |  |  |
| **Tumor Location** |  |  |  |  |  |
| **Proximal colon** | 38 (25.0) | 33 (25.0) | 3 (100.0)) | 2 (50.0) | 0.009 |
| **Distal colon or rectum** | 114 (75.0) | 99 (75.0) | 0 (0.0) | 2 (50.0) |  |
|  |  |  |  |  |  |
| **Tumor (T)** |  |  |  |  |  |
| **Tis/T1/T2** | 53 (34.9) | 42 (31.8) | 0 (0.0) | 2 (50.0) | 0.365 |
| **T3/T4 (a b)** | 99 (65.1) | 90 (68.2) | 3 (100.0)) | 2 (50.0) |  |
|  |  |  |  |  |  |
| **Clinical stage** |  |  |  |  |  |
| **E0/I** | 44 (28.9) | 35 (26.5) | 0 (0.0) | 2 (50.0) | 0.633 |
| **EII/III** | 102 (67.1) | 91 (68.9) | 3 (100.0)) | 2 (50.0) |  |
| **EIV** | 6 (3.9) | 6 (4.5) | 0 (0.0) | 0 (0.0) |  |
|  |  |  |  |  |  |
| **Tumor differentiation** |  |  |  |  |  |
| **Well to moderate** | 139 (92.7) | 121 (92.4) | 3 (100.0) | 2 (66.7) | 0.236 |
| **Poor** | 11 (7.3) | 10 (7.7) | 0 (0.0) | 1 (33.3) |  |
|  |  |  |  |  |  |
| **MSI status*** |  |  |  |  |  |
| **MSI-negative** | 131 (86.2) | 116 (87.9) | 2 (50.0) | 0 (0.0) | <0.0001 |
| **MSI-positive** | 21 (13.8) | 16 (12.1) | 2 (50.0) | 3 (100.0) |  |
|  |  |  |  |  |  |
| ***BRAF* mutation#** |  |  |  |  |  |
| **Mutant** | 11 (7.3) | 8 (6.1) | 2 (50.0) | 1 (33.3) | 0.002 |
| **Wild type** | 140 (92.7) | 123 (93.9) | 2 (50.0) | 2 (66.7) |  |
|  |  |  |  |  |  |
| **MLH1 protein expression*** |  |  |  |  |  |
| **Positive** | 117 (87.3) | 103 (89.6) | 2 (50.0) | 0 (0.0) | <0.0001 |
| **Negative** | 17 (12.7) | 12 (10.4) | 2 (50.0) | 3 (100.0) |  |
|  |  |  |  |  |  |
| **MSH2 protein expression*** |  |  |  |  |  |
| **Positive** | 131 (97.8) | 112 (97.4) | 3 (100.0) | 4 (100.0) | 0.911 |
| **Negative** | 3 (2.2) | 3 (2.6) | 0 (0.0) | 0 (0.0) |  |
|  |  |  |  |  |  |
| **MSH6 protein expression*** |  |  |  |  |  |
| **Positive** | 133 (99.3) | 114 (99.1) | 3 (100.0) | 4 (100.0) | 0.970 |
| **Negative** | 1 (0.7) | 1 (0.9) | 0 (0.0) | 0 (0.0) |  |
|  |  |  |  |  |  |
| **PMS2 protein expression*** |  |  |  |  |  |
| **Positive** | 119 (88.8) | 105 (91.3) | 2 (50.0) | 0 (0.0) | <0.0001 |
| **Negative** | 15 (11.2) | 10 (8.7) | 2 (50.0) | 3 (100.0) |  |

**Percentage indicates the proportion of cases with a specific clinical, pathological or molecular variable according to the amount of *F. nucleatum* DNA in colorectal cancer tissue. †To assess associations between the ordinal categories (negative, low and high) of the amount of *F. nucleatum* DNA in colorectal cancer tissue and categorical variables, Fisher’s exact test was performed. # previously reported Berardinelli et al. 2019; $ unpublished data; MSI, microsatellite instability.**

**Supplementary Table 4 -** Classification of the 152 cases included in this study regarding the presence and level of Fusobacterium nucleatum in the tumor tissue (fresh frozen or paraffin embedded) and normal adjacent tissue (fresh frozen). FFT = fresh frozen tissue; FFPE = formalin fixed paraffin embedded; NA = normal adjacent tissue.

| Patient number | Fusobacterium_FFT | Fuso_quant_3cat_FFT | Fusobacterium_NA | Fuso_quant_3cat_NA | Fusobacterium_FFPE | Fuso_quant_3cat_FFPE |
| --- | --- | --- | --- | --- | --- | --- |
| 1 | negative | negative | not evaluated | not evaluated | negative | negative |
| 2 | negative | negative | not evaluated | not evaluated | negative | negative |
| 3 | positive | low | not evaluated | not evaluated | negative | negative |
| 4 | negative | negative | not evaluated | not evaluated | negative | negative |
| 5 | negative | negative | not evaluated | not evaluated | negative | negative |
| 6 | negative | negative | not evaluated | not evaluated | negative | negative |
| 7 | negative | negative | not evaluated | not evaluated | negative | negative |
| 8 | negative | negative | not evaluated | not evaluated | negative | negative |
| 9 | negative | negative | not evaluated | not evaluated | negative | negative |
| 10 | negative | negative | not evaluated | not evaluated | negative | negative |
| 11 | positive | low | not evaluated | not evaluated | negative | negative |
| 12 | negative | negative | not evaluated | not evaluated | negative | negative |
| 13 | negative | negative | not evaluated | not evaluated | negative | negative |
| 14 | negative | negative | not evaluated | not evaluated | negative | negative |
| 15 | negative | negative | not evaluated | not evaluated | negative | negative |
| 16 | negative | negative | not evaluated | not evaluated | negative | negative |
| 17 | negative | negative | not evaluated | not evaluated | negative | negative |
| 18 | negative | negative | not evaluated | not evaluated | negative | negative |
| 19 | negative | negative | not evaluated | not evaluated | negative | negative |
| 20 | negative | negative | not evaluated | not evaluated | negative | negative |
| 21 | negative | negative | not evaluated | not evaluated | negative | negative |
| 22 | negative | negative | not evaluated | not evaluated | negative | negative |
| 23 | negative | negative | not evaluated | not evaluated | negative | negative |
| 24 | negative | negative | not evaluated | not evaluated | negative | negative |
| 25 | positive | low | not evaluated | not evaluated | negative | negative |
| 26 | negative | negative | not evaluated | not evaluated | negative | negative |
| 27 | positive | low | not evaluated | not evaluated | negative | negative |
| 28 | negative | negative | not evaluated | not evaluated | negative | negative |
| 29 | positive | high | not evaluated | not evaluated | negative | negative |
| 30 | negative | negative | not evaluated | not evaluated | negative | negative |
| 31 | negative | negative | not evaluated | not evaluated | negative | negative |
| 32 | negative | negative | not evaluated | not evaluated | negative | negative |
| 33 | negative | negative | not evaluated | not evaluated | negative | negative |
| 34 | negative | negative | not evaluated | not evaluated | negative | negative |
| 35 | negative | negative | not evaluated | not evaluated | negative | negative |
| 36 | negative | negative | not evaluated | not evaluated | negative | negative |
| 37 | positive | low | not evaluated | not evaluated | negative | negative |
| 38 | negative | negative | not evaluated | not evaluated | negative | negative |
| 39 | negative | negative | not evaluated | not evaluated | negative | negative |
| 40 | negative | negative | not evaluated | not evaluated | negative | negative |
| 41 | negative | negative | not evaluated | not evaluated | negative | negative |
| 42 | negative | negative | not evaluated | not evaluated | negative | negative |
| 43 | negative | negative | not evaluated | not evaluated | negative | negative |
| 44 | negative | negative | not evaluated | not evaluated | negative | negative |
| 45 | negative | negative | not evaluated | not evaluated | negative | negative |
| 46 | negative | negative | not evaluated | not evaluated | negative | negative |
| 47 | negative | negative | not evaluated | not evaluated | negative | negative |
| 48 | positive | high | not evaluated | not evaluated | negative | negative |
| 49 | negative | negative | not evaluated | not evaluated | negative | negative |
| 50 | positive | low | not evaluated | not evaluated | negative | negative |
| 51 | negative | negative | not evaluated | not evaluated | negative | negative |
| 52 | negative | negative | not evaluated | not evaluated | negative | negative |
| 53 | negative | negative | not evaluated | not evaluated | negative | negative |
| 54 | negative | negative | not evaluated | not evaluated | negative | negative |
| 55 | negative | negative | not evaluated | not evaluated | negative | negative |
| 56 | negative | negative | not evaluated | not evaluated | negative | negative |
| 57 | positive | high | not evaluated | not evaluated | positive | high |
| 58 | negative | negative | not evaluated | not evaluated | negative | negative |
| 59 | negative | negative | not evaluated | not evaluated | negative | negative |
| 60 | negative | negative | not evaluated | not evaluated | negative | negative |
| 61 | negative | negative | not evaluated | not evaluated | negative | negative |
| 62 | negative | negative | not evaluated | not evaluated | negative | negative |
| 63 | negative | negative | not evaluated | not evaluated | negative | negative |
| 64 | negative | negative | not evaluated | not evaluated | negative | negative |
| 65 | negative | negative | not evaluated | not evaluated | negative | negative |
| 66 | negative | negative | not evaluated | not evaluated | negative | negative |
| 67 | positive | low | not evaluated | not evaluated | negative | negative |
| 68 | negative | negative | not evaluated | not evaluated | negative | negative |
| 69 | negative | negative | not evaluated | not evaluated | negative | negative |
| 70 | negative | negative | negative | low/negative | negative | negative |
| 71 | positive | low | negative | low/negative | negative | negative |
| 72 | negative | negative | negative | low/negative | negative | negative |
| 73 | negative | negative | not evaluated | not evaluated | negative | negative |
| 74 | negative | negative | not evaluated | not evaluated | negative | negative |
| 75 | positive | high | not evaluated | not evaluated | negative | negative |
| 76 | negative | negative | positive | high | negative | negative |
| 77 | negative | negative | not evaluated | not evaluated | negative | negative |
| 78 | positive | high | not evaluated | not evaluated | negative | negative |
| 79 | negative | negative | not evaluated | not evaluated | negative | negative |
| 80 | negative | negative | not evaluated | not evaluated | negative | negative |
| 81 | negative | negative | negative | low/negative | negative | negative |
| 82 | positive | low | not evaluated | not evaluated | negative | negative |
| 83 | positive | high | not evaluated | not evaluated | negative | negative |
| 84 | positive | low | negative | low/negative | negative | negative |
| 85 | negative | negative | not evaluated | not evaluated | negative | negative |
| 86 | negative | negative | not evaluated | not evaluated | negative | negative |
| 87 | negative | negative | negative | low/negative | negative | negative |
| 88 | negative | negative | not evaluated | not evaluated | negative | negative |
| 89 | negative | negative | not evaluated | not evaluated | not evaluated | not evaluated |
| 90 | positive | low | positive | high | negative | negative |
| 91 | negative | negative | negative | low/negative | negative | negative |
| 92 | negative | negative | not evaluated | not evaluated | negative | negative |
| 93 | positive | high | not evaluated | not evaluated | not evaluated | not evaluated |
| 94 | negative | negative | negative | low/negative | not evaluated | not evaluated |
| 95 | negative | negative | negative | low/negative | negative | negative |
| 96 | negative | negative | not evaluated | not evaluated | negative | negative |
| 97 | negative | negative | negative | low/negative | negative | negative |
| 98 | negative | negative | negative | low/negative | not evaluated | not evaluated |
| 99 | negative | negative | negative | low/negative | not evaluated | not evaluated |
| 100 | negative | negative | negative | low/negative | not evaluated | not evaluated |
| 101 | negative | negative | negative | low/negative | negative | negative |
| 102 | negative | negative | not evaluated | not evaluated | not evaluated | not evaluated |
| 103 | negative | negative | negative | low/negative | negative | negative |
| 104 | negative | negative | not evaluated | not evaluated | negative | negative |
| 105 | negative | negative | negative | low/negative | negative | negative |
| 106 | negative | negative | negative | low/negative | negative | negative |
| 107 | positive | high | positive | high | negative | negative |
| 108 | negative | negative | negative | low/negative | negative | negative |
| 109 | negative | negative | negative | low/negative | not evaluated | not evaluated |
| 110 | negative | negative | not evaluated | not evaluated | not evaluated | not evaluated |
| 111 | negative | negative | negative | low/negative | negative | negative |
| 112 | negative | negative | negative | low/negative | not evaluated | not evaluated |
| 113 | negative | negative | negative | low/negative | not evaluated | not evaluated |
| 114 | negative | negative | negative | low/negative | not evaluated | not evaluated |
| 115 | negative | negative | not evaluated | not evaluated | negative | negative |
| 116 | negative | negative | not evaluated | not evaluated | not evaluated | not evaluated |
| 117 | negative | negative | not evaluated | not evaluated | negative | negative |
| 118 | negative | negative | not evaluated | not evaluated | negative | negative |
| 119 | negative | negative | not evaluated | not evaluated | negative | negative |
| 120 | negative | negative | not evaluated | not evaluated | negative | negative |
| 121 | negative | negative | not evaluated | not evaluated | negative | negative |
| 122 | negative | negative | not evaluated | not evaluated | negative | negative |
| 123 | negative | negative | negative | low/negative | negative | negative |
| 124 | positive | high | not evaluated | not evaluated | negative | negative |
| 125 | negative | negative | not evaluated | not evaluated | negative | negative |
| 126 | negative | negative | not evaluated | not evaluated | positive | high |
| 127 | negative | negative | negative | low/negative | negative | negative |
| 128 | negative | negative | not evaluated | not evaluated | negative | negative |
| 129 | negative | negative | not evaluated | not evaluated | negative | negative |
| 130 | negative | negative | not evaluated | not evaluated | negative | negative |
| 131 | positive | low | not evaluated | not evaluated | negative | negative |
| 132 | negative | negative | not evaluated | not evaluated | negative | negative |
| 133 | negative | negative | not evaluated | not evaluated | negative | negative |
| 134 | positive | low | not evaluated | not evaluated | positive | low |
| 135 | negative | negative | not evaluated | not evaluated | negative | negative |
| 136 | positive | high | not evaluated | not evaluated | positive | low |
| 137 | negative | negative | not evaluated | not evaluated | negative | negative |
| 138 | positive | high | not evaluated | not evaluated | negative | negative |
| 139 | positive | low | negative | low/negative | negative | negative |
| 140 | positive | high | negative | low/negative | negative | negative |
| 141 | positive | high | not evaluated | not evaluated | negative | negative |
| 142 | negative | negative | not evaluated | not evaluated | negative | negative |
| 143 | positive | high | not evaluated | not evaluated | negative | negative |
| 144 | negative | negative | not evaluated | not evaluated | negative | negative |
| 145 | positive | low | not evaluated | not evaluated | negative | negative |
| 146 | positive | high | not evaluated | not evaluated | positive | low |
| 147 | negative | negative | not evaluated | not evaluated | negative | negative |
| 148 | positive | low | not evaluated | not evaluated | positive | high |
| 149 | positive | high | not evaluated | not evaluated | negative | negative |
| 150 | positive | low | not evaluated | not evaluated | negative | negative |
| 151 | positive | high | not evaluated | not evaluated | negative | negative |
| 152 | positive | high | not evaluated | not evaluated | positive | high |
